# Assay-Specific Diagnostic Thresholds for Adrenal Insufficiency: A Retrospective Comparison of Monoclonal Cortisol Measurements Using Roche Elecsys Cortisol II and Tosoh AIA-CL2400

**DOI:** 10.7759/cureus.107059

**Published:** 2026-04-14

**Authors:** Shunsuke Kato, Hana Akanuma, Mitsuhiko Nara, Takehiro Sato, Tsukasa Morii, Hiroki Fujita, Hironori Waki

**Affiliations:** 1 Center for Medical Education and Training, Akita University Hospital, Akita, JPN; 2 Department of Metabolism and Endocrinology, Akita University Graduate School of Medicine, Akita, JPN; 3 Regional Collaborative Department of Oga Namahage Community Medicine, Akita University Graduate School of Medicine, Akita, JPN; 4 Department of Advanced Medical Research Promotion, Akita University Graduate School of Medicine, Akita, JPN; 5 Center for Physician Scientist Training, Akita University Graduate School of Medicine, Akita, JPN

**Keywords:** adrenal insufficiency, cortisol measurement, cut-off values, endocrine, monoclonal antibody

## Abstract

Background

The diagnosis of adrenal insufficiency traditionally relies on peak cortisol values during stimulation testing, with a cut-off of 18 µg/dL established using polyclonal antibody assays. However, newer monoclonal antibody-based assays show reduced cross-reactivity with other glucocorticoids. In April 2022, our hospital changed the cortisol measurement method from Roche Elecsys Cortisol II to Tosoh AIA-CL2400, both using monoclonal antibodies but with potentially different measurement characteristics. In addition to stimulation test results, physicians initiate hydrocortisone replacement based on the patient’s clinical condition.

Methods

We retrospectively reviewed 226 patients who underwent adrenocorticotropic hormone (ACTH) or corticotropin-releasing hormone (CRH) stimulation testing for suspected adrenal insufficiency between March 2016 and September 2023. Patients with baseline cortisol ≥18 µg/dL or receiving steroid medications other than hydrocortisone were excluded. Assessment variables included baseline cortisol, baseline ACTH, peak cortisol, and the change in cortisol during stimulation tests. Clinical diagnosis of adrenal insufficiency was defined by initiation of hydrocortisone replacement therapy. Receiver operating characteristic (ROC) curve analysis was performed to determine optimal cut-off values. Background factors included gender, age, presence of adrenal insufficiency symptoms, and structural or functional abnormalities of the hypothalamus-pituitary-adrenal (HPA) axis. Simple and multiple logistic regression analyses were conducted with the diagnosis of adrenal insufficiency as the dependent variable.

Results

The mean age was 54 years, and 75 (33.2%) of the participants were male. Adrenal insufficiency symptoms were present in 118 (52.2%), while 135 (59.7%) had either structural or functional abnormalities of the HPA axis. When comparing values between the two assay periods, peak cortisol was significantly different (mean: 14.6 vs. 16.8 µg/dL for Elecsys Cortisol II vs. AIA-CL2400). The proportion of patients with cortisol peaks <18 µg/dL differed (128 (68.1%) vs. 18 (47.4%)), but hydrocortisone initiation remained similar (82 (43.6%) vs. 14 (36.8%)). Multiple logistic regression analysis identified peak cortisol, presence of symptoms, and structural or functional abnormalities of the HPA axis as significant factors associated with the clinical diagnosis. ROC curve analysis revealed that the optimal cut-off value increased from 14.5 µg/dL before the assay change to 17.0 µg/dL after the change. When stratified by symptom presence, cut-off values were 14.5 µg/dL (with symptoms) and 13.1 µg/dL (without symptoms) before the change, and 17.0 µg/dL (with symptoms) and 15.1 µg/dL (without symptoms) after the change. When stratified by structural or functional abnormalities of the HPA axis, cut-off values were 13.7 µg/dL (with abnormalities) and 14.5 µg/dL (without abnormalities) before the change, and 17.5 µg/dL (with abnormalities) and 16.4 µg/dL (without abnormalities) after the change.

Conclusion

Our study provides two key messages: First, differences in assay methodology can significantly alter cortisol values and appropriate diagnostic cut-offs, even when transitioning between monoclonal antibody-based assays. Second, both peak cortisol and symptom presence were crucial for clinical diagnosis of adrenal insufficiency, with potentially lower cut-off values being appropriate for asymptomatic cases.

## Introduction

The most important clinical scenario for the measurement of cortisol is during the diagnosis of adrenal insufficiency. While insulin stimulation tests or adrenocorticotropic hormone (ACTH) stimulation tests have been traditionally used, the former carries high risks, making the latter the current gold standard for diagnosis [1]. The cut-off value for peak cortisol during ACTH stimulation testing has traditionally been established at 18 µg/dL based on the older polyclonal antibody assays [2]. In the Endocrine Society Clinical Practice Guideline for the Diagnosis and Treatment of Primary Adrenal Insufficiency, co-sponsored by the European Society of Endocrinology and the American Association for Clinical Chemistry, a peak cortisol level <18 µg/dL (500 nmol/L) is defined as the diagnostic threshold, although this cut-off is noted to be assay-dependent [3]. The Japan Endocrine Society’s guidelines have similarly adopted the 18 µg/dL threshold (with 15 µg/dL for primary adrenal insufficiency) [4].

Currently, assays using monoclonal antibodies, which offer greater specificity than polyclonal antibodies, are more commonly employed. These newer assays have been reported to yield lower values compared to older assays due to reduced cross-reactivity with glucocorticoids other than cortisol. For instance, studies using Roche Elecsys Cortisol II with monoclonal antibodies have reported cut-off values for cortisol after ACTH stimulation testing in the range of 12.7-15.7 µg/dL [5-11]. Similarly, reports using Beckman Access with monoclonal antibodies have documented cut-off values ranging from 13.2 to 14.7 µg/dL [8,12].

In April 2022, the cortisol measurement assay at our hospital was changed from Roche Elecsys Cortisol II to Tosoh AIA-CL2400. The former employs a sheep monoclonal antibody electrochemiluminescence immunoassay (ECLIA) [13], and the latter employs a mouse monoclonal antibody chemiluminescent enzyme immunoassay (CLEIA) developed in Japan [14]. The cross-reactivity with cortisone (a predominant interfering steroid) was 5.1% and 2.3% for the two assays, respectively [14]. Thus, despite both methods using monoclonal antibodies, they may exhibit different measurement characteristics. While there have been reports comparing assays using polyclonal and monoclonal antibodies, there have been few studies comparing different monoclonal antibody assays with each other [15].

In addition to the aforementioned assay-related issues, endocrinologists in clinical practice evaluate the overall clinical presentation of each patient when deciding whether to initiate oral hydrocortisone replacement. In order to elucidate how the change in the cortisol measurement methods and clinical judgment by endocrinologists affected overall diagnosis, we retrospectively investigated the diagnostic rate of adrenal insufficiency before and after the implementation of the new cortisol assay at our hospital, and how the thresholds are different from the established 18 µg/dL for the clinical diagnosis of adrenal insufficiency. In order to address these questions, the specific objectives of this study were twofold: (1) to compare peak cortisol values and assay-specific diagnostic cut-off values between the two assay periods using receiver operating characteristic (ROC) curve analysis and (2) to identify clinical factors independently associated with the diagnosis of adrenal insufficiency using logistic regression analysis. 

## Materials and methods

We retrospectively reviewed cases in which a rapid ACTH stimulation test and/or corticotropin-releasing hormone (CRH) stimulation test was performed for the diagnosis of adrenal insufficiency at Akita University Hospital's Department of Diabetes and Endocrinology, in both outpatient and inpatient settings, between March 2016 and September 2023. The decision to perform the stimulation test and the clinical diagnosis of adrenal insufficiency was made by one or more physicians specializing in diabetes and endocrinology.

Both stimulation tests were performed after patients arrived at 8:30 AM in a fasting state and rested in bed for 30 minutes prior to the start of testing. For the rapid ACTH stimulation test, 250 µg of tetracosactide was administered intravenously, with cortisol sampling at 0, 30, and 60 minutes. For the CRH stimulation test, 100 µg of human CRH was administered intravenously, with cortisol sampling at 0, 30, 60, and 90 minutes. Assessment variables included baseline cortisol, baseline ACTH, peak cortisol (defined as the highest cortisol value recorded at any time point during each stimulation test), and the change in cortisol during the stimulation test. Clinical diagnosis of adrenal insufficiency was defined as initiation of hydrocortisone replacement therapy, as determined by the attending diabetes and endocrinology specialist based on a comprehensive clinical assessment, including peak cortisol values, the presence of adrenal insufficiency symptoms, and structural or functional abnormalities of the HPA axis.

In our study, Roche Elecsys Cortisol II (electrochemiluminescence immunoassay, ECLIA) was used from March 2016 to March 2022, and Tosoh AIA-CL2400 (chemiluminescent enzyme immunoassay, CLEIA) was used from April 2022 to September 2023. Assessment variables were compared before and after April 2022, which marked the timing of the change in measurement assay. Background factors assessed included gender, age at the time of testing, presence of adrenal insufficiency symptoms, and structural or functional abnormalities of the HPA axis. Cases with a baseline cortisol of ≥18 µg/dL and those treated with steroids other than hydrocortisone were excluded.

ROC curve analysis was performed to determine the optimal cut-off value of peak cortisol for the diagnosis of adrenal insufficiency. Additional ROC analyses were conducted separately based on the presence of symptoms and structural or functional abnormalities of the HPA axis. Differences between the two cortisol measurement assay groups were analyzed using the Mann-Whitney U test. Simple and multiple logistic regression analyses were performed with the diagnosis of adrenal insufficiency as the dependent variable. GraphPad Prism 8 (Dotmatics, Boston, MA, USA) was used for statistical analysis, with p < 0.05 considered statistically significant. Data visualization was performed using GraphPad Prism 8 and PowerPoint 2016 (Microsoft Corp., Redmond, WA, USA).

## Results

**Figure 1:**
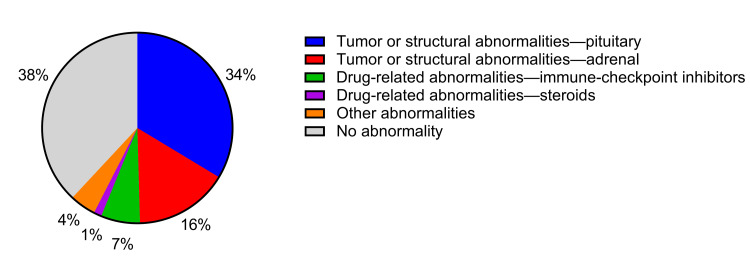
Overview of general structural or functional abnormalities of the HPA axis HPA axis: hypothalamic-pituitary-adrenal axis.

After excluding five patients with baseline cortisol levels >18 µg/dL and four patients receiving steroid medications other than hydrocortisone, a total of 226 patients were included in the analysis. Of these, 151 (66.8%) and 75 (33.2%) patients underwent ACTH and CRH stimulation tests, respectively. The mean age was 54 years, and 75 (33.2%) of the participants were male. Adrenal insufficiency symptoms were present in 118 (52.2%) patients, while 135 (59.7%) had structural or functional abnormalities of the HPA axis (Table 1). The distribution of these abnormalities is shown in Table 2 and Figure 1. Tumor or structural abnormalities of the pituitary were the most common (33.6%), followed by those of the adrenal (15.9%). Drug-related abnormalities due to immune-checkpoint inhibitors accounted for 6.6%, which may be lower than currently observed in clinical practice. Steroid-related abnormalities were less frequent (1.3%). Other abnormalities accounted for 4.4%.

**Table 1:** Background characteristics of the patients ACTH: adrenocorticotropic hormone, CRH: corticotropin-releasing hormone, HPA axis: hypothalamic-pituitary-adrenal axis.

Variable	Value
ACTH stimulation test, n (%)	151 (66.8%)
CRH stimulation test, n (%)	75 (33.2%)
Age (years, mean ± SD)	54.0 ± 16
Gender (men), n (%)	75 (33.2%)
Symptoms present, n (%)	118 (52.2%)
Structural or functional abnormalities of the HPA axis, n (%)	135 (59.7%)
Assay: Roche Elecsys Cortisol II, n (%)	188 (83.2%)
Assay: Tosoh AIA-CL2400, n (%)	38 (16.8%)

**Table 2:** General and specific etiologies of structural or functional abnormalities of the HPA axis ACTH: adrenocorticotropic hormone, irAE: immune-related adverse events, HPA axis: hypothalamic-pituitary-adrenal axis.

Type of abnormality	Anatomical site/subtype	Clinical condition	N	(%)
Tumor or structural abnormalities	Pituitary		76	33.60%
		Post-pituitary surgery	33	14.60%
		Pituitary tumor	26	11.50%
		Other cranial tumors	13	5.75%
		Pituitary enlargement	2	0.88%
		Pituitary hemorrhage	1	0.44%
		Acromegaly	1	0.44%
	Adrenal		36	15.90%
		Post-adrenalectomy	23	10.20%
		Adrenal tumor	10	4.42%
		Adrenal metastatic cancer	2	0.88%
		Adrenal infiltration	1	0.44%
Drug-related abnormalities	Immune-checkpoint inhibitors		15	6.64%
		History of immune-checkpoint inhibitor therapy	7	3.10%
		irAE (pituitary)	5	2.21%
		irAE (adrenal)	3	1.33%
	Steroids	History of exogenous steroid use	3	1.33%
Other abnormalities			10	4.41%
		Other hormone abnormality	5	2.21%
		Isolated ACTH deficiency	2	0.88%
		Pituitary genetic disorder	2	0.88%
		Other pituitary abnormality	1	0.44%
No abnormality		No abnormality	86	38.10%

The remaining patients had symptoms or relevant medical histories and were identified during investigations for electrolyte abnormalities, secondary hypertension, or secondary obesity.

**Figure 2:**
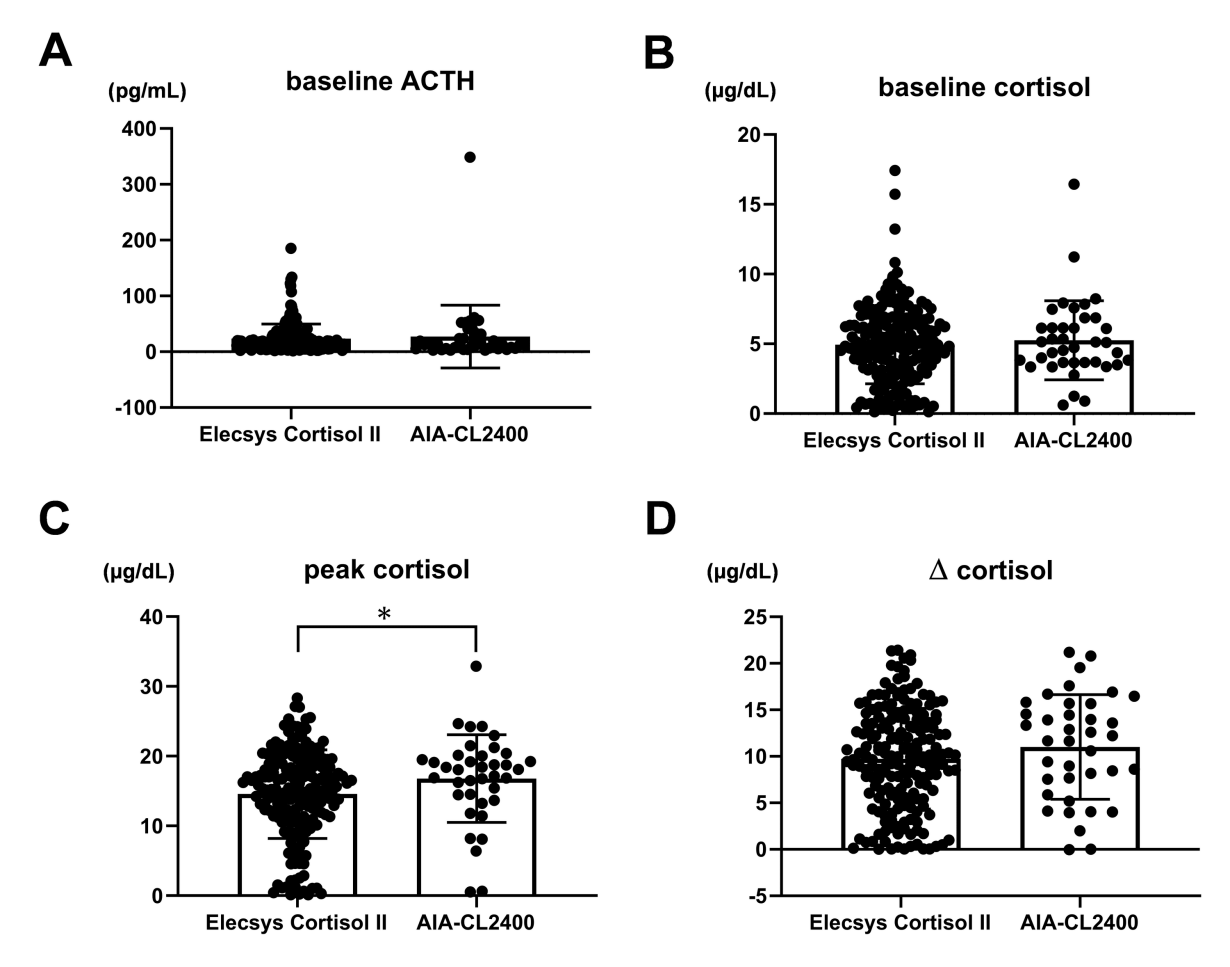
Comparison of stimulation test values measured by Roche Elecsys Cortisol II and Tosoh AIA-CL2400. Baseline ACTH (A), baseline cortisol (B), peak cortisol (C), and increase in cortisol levels (D) during ACTH- or CRH-stimulation tests Bars represent mean ± SD. *p < 0.05 (Mann-Whitney U = 2800, p = 0.035 (two-tailed)).

Cortisol levels of 188 (83.2%) cases were measured by Roche Elecsys Cortisol II, and those of the 38 (16.8%) cases were measured by Tosoh AIA-CL2400 (Table 1). Figure 2 illustrates the distribution of values obtained during ACTH or CRH stimulation tests using these measurement methods.

Peak cortisol values during the stimulation tests differed significantly between the two methods (mean value: 14.6 vs. 16.8 µg/dL for Roche Elecsys Cortisol II and Tosoh AIA-CL2400, respectively; Mann-Whitney U = 2800, two-tailed p = 0.035).

**Figure 3:**
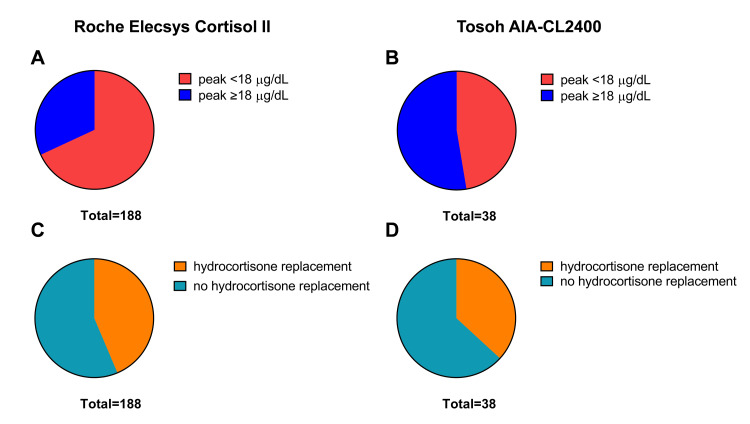
Proportion of patients with peak cortisol levels <18 μg/dL (A, B) and receiving hydrocortisone replacement (C, D), stratified by assay: Roche Elecsys Cortisol II (A, C) and Tosoh AIA-CL2400 (B, D)

The proportion of patients with cortisol peaks below 18 µg/dL was 128 (68.1%) in the Roche Elecsys Cortisol II assay, and 18 (47.4%) in the Tosoh AIA-CL2400 assay, representing a significant difference. In contrast, the proportion of clinical diagnoses of adrenal insufficiency judged by the initiation of hydrocortisone replacement was 82 (43.6%) and 14 (36.8%), respectively, which were relatively similar in the two groups (Figure 3).

These results suggest that, although 18 µg/dL has been considered the gold standard for diagnosing adrenal insufficiency, endocrinologists initiate hydrocortisone replacement at similar rates without relying solely on the 18 µg/dL threshold.

**Figure 4:**
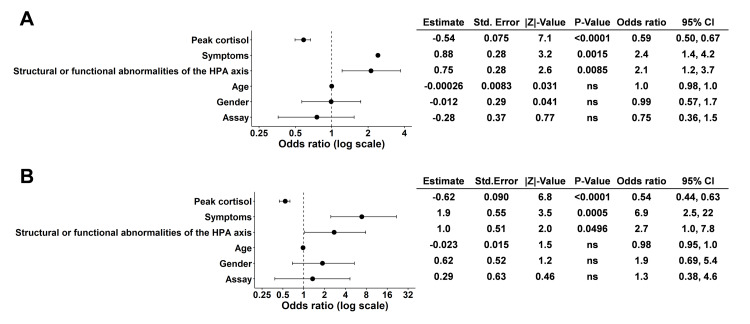
Simple logistic regression analysis (A) and multiple logistic regression analysis (B) for the clinical diagnosis of adrenal insufficiency

To identify factors contributing to the clinical diagnosis of adrenal insufficiency, we performed simple logistic regression analysis (Figure 4A).

Among the variables examined, peak cortisol levels (odds ratio = 0.59, 95% CI: 0.50, 0.67), the presence of symptoms (odds ratio = 2.4, 95% CI: 1.4, 4.2), and structural or functional abnormalities of the HPA axis (odds ratio = 2.1, 95% CI: 1.2, 3.7) showed significant associations (Figure 4A). To elucidate the independent contributions of these factors to the clinical diagnosis of adrenal insufficiency, we performed multiple logistic regression analysis including these variables (Figure 4B). Peak cortisol levels (odds ratio = 0.54, 95% CI: 0.44, 0.63), the presence of symptoms (odds ratio = 6.9, 95% CI: 2.5, 22), and structural or functional abnormalities of the HPA axis (odds ratio = 2.7, 95% CI: 1.0, 7.8) showed independent significant associations with the diagnosis (Figure 4B).

**Figure 5:**
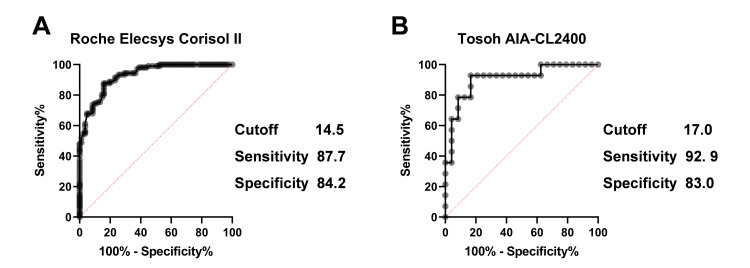
Receiver operating characteristic (ROC) curve analysis of clinical diagnosis of adrenal insufficiency in Roche Elecsys Cortisol II (A) and Tosoh AIA-CL2400 (B) assays

Finally, we investigated the optimal peak cortisol cut-off value for the clinical diagnosis of adrenal insufficiency for each measurement assay. ROC curve analysis revealed that the optimal cut-off value was 14.5 µg/dL for the Roche Elecsys Cortisol II assay, and 17.0 µg/dL for the Tosoh AIA-CL2400 assay (Figure 5).

**Figure 6:**
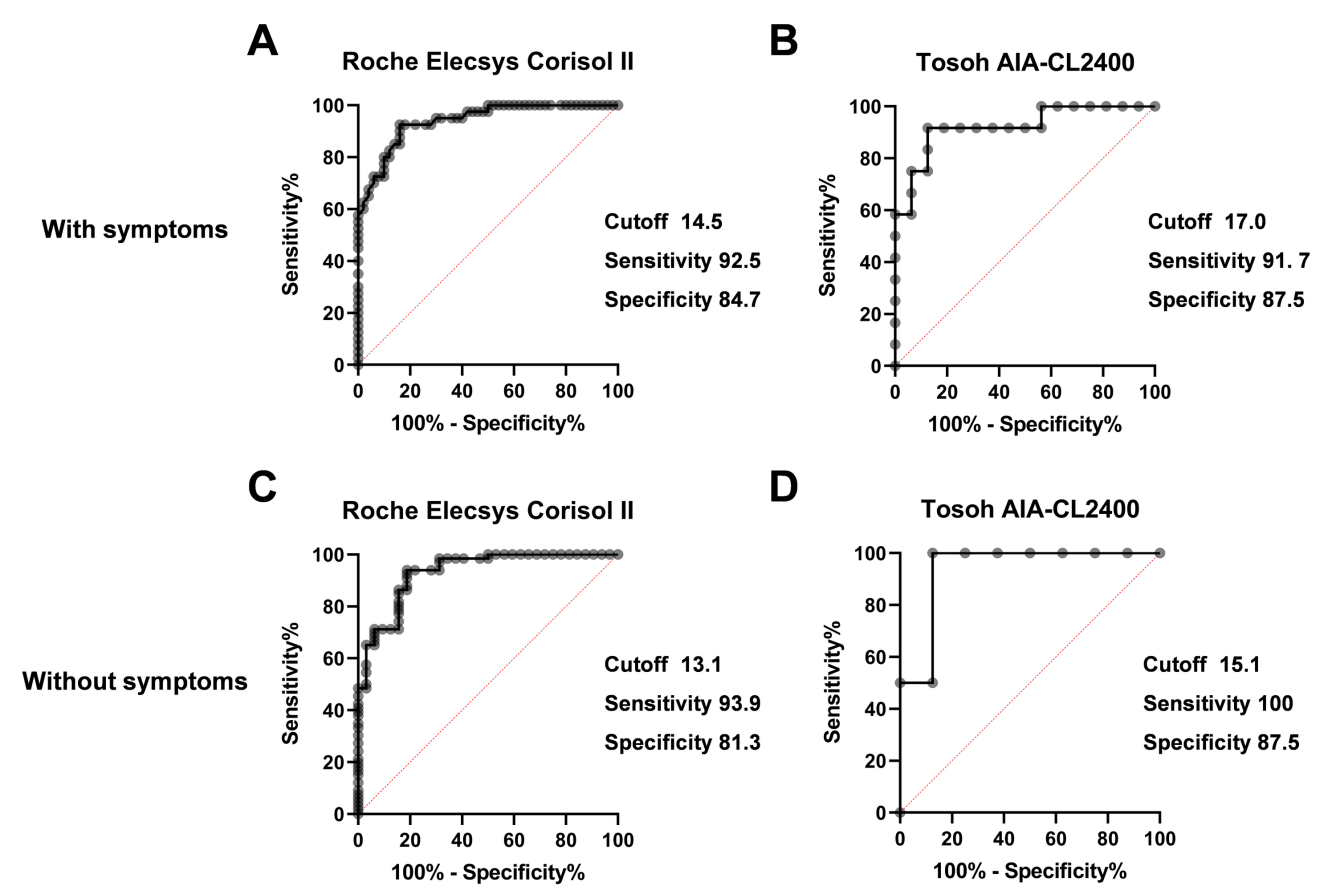
ROC analysis for the clinical diagnosis of adrenal insufficiency using the Roche Elecsys Cortisol II (A, C) and Tosoh AIA-CL2400 (B, D) assays in patients with symptoms (A, B) and without symptoms (C, D)

We also examined the cut-off values separately based on the presence of symptoms and structural or functional abnormalities of the HPA axis. The cut-off value in the presence of symptoms was 14.5 µg/dL, whereas the cut-off value in the absence of symptoms was 13.1 µg/dL for the Roche Elecsys Cortisol II assay (Figures 6A, 6B).

**Figure 7:**
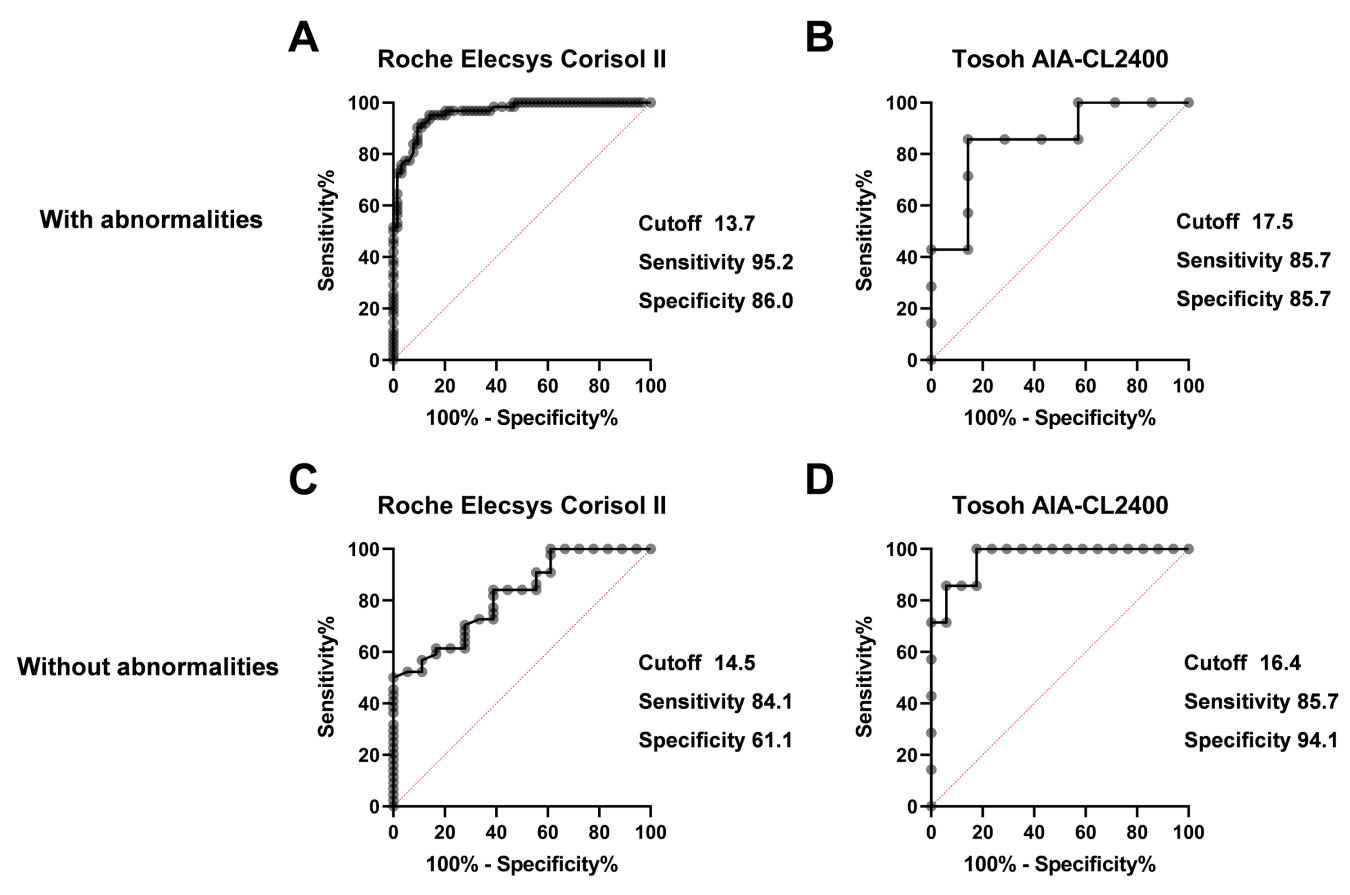
ROC analysis for the clinical diagnosis of adrenal insufficiency using the Roche Elecsys Cortisol II (A, C) and Tosoh AIA-CL2400 (B, D) assays in patients with (A, B) or without structural or functional abnormalities of the HPA axis (C, D)

The cut-off values were 17.0 µg/dL in the presence of symptoms and 15.1 µg/dL in the absence of symptoms for the Tosoh AIA-CL2400 assay (Figures 6C, 6D). The cut-off values were 13.7 µg/dL for cases with structural or functional abnormalities of the HPA axis and 14.5 µg/dL for those without abnormalities for the Roche Elecsys Cortisol II assay (Figures 7A, 7B).

The cut-off values were 17.5 µg/dL for cases with structural or functional abnormalities of the HPA axis and 16.4 µg/dL for those without for the Tosoh AIA-CL2400 assay (Figures 7C, 7D).

## Discussion

Differences between assays using polyclonal and monoclonal antibodies have been previously documented, with monoclonal antibody-based assays typically yielding values 20%-30% lower than those using polyclonal antibodies [7-10]. However, the American/European and Japanese guidelines consistently apply a cut-off value of 18 µg/dL, which presents an important clinical issue. Additionally, research comparing different monoclonal antibody-based assays remains insufficient. This creates a potential pitfall in which differences among monoclonal antibody assays might be overlooked. Such misinterpretation may lead to misclassification in patients on the borderline of adrenal insufficiency, resulting in some receiving unnecessary treatment, while others who require treatment may not receive it.

The correlation between the assays used in this study was previously reported as y = 1.08x + 0.97 (µg/dL) [14]. When we substitute 14.5 µg/dL (our pre-change cut-off value) for x in this equation, we obtain y = 16.6 µg/dL, which closely approximates the actual value of 17.0 µg/dL obtained in our analysis. This consistency enhances the validity of our findings. As demonstrated in the basic study, our study confirmed that cortisol values were higher after the assay change.

In general, it is assumed that newer assays using antibodies with improved accuracy and reduced cross-reactivity with other steroids tend to yield lower values. However, in our case, the cross-reactivity with prednisolone was lower in the Roche assay (7.7% in the Roche Elecsys Cortisol II assay vs. 9.6% in the Tosoh AIA-CL2400 assay) [14]. This represents a second potential pitfall in interpretation. Standardization of assays would help resolve these issues.

Interestingly, although we observed a significant difference in peak cortisol values before and after the change in measurement assay, there was no corresponding difference in the actual rate of clinical diagnosis of adrenal insufficiency. This suggests that clinicians do not rely solely on absolute peak cortisol values when diagnosing adrenal insufficiency. The results of our multiple logistic regression analysis revealed that, in addition to peak cortisol, the most important factors influencing the diagnosis of adrenal insufficiency were the presence of symptoms and structural or functional abnormalities of the HPA axis.

It is also noteworthy that ROC curve analysis of the two groups stratified by symptom presence showed differences in optimal cut-off values: the cut-off value for clinical diagnosis of adrenal insufficiency was lower in asymptomatic cases. Similarly, the differences in cut-off values were observed when stratified by the presence of structural or functional abnormalities of the HPA axis. These results suggest that, in the absence of symptoms or structural or functional abnormalities of the HPA axis, it may be appropriate to adjust the cut-off values used for diagnosing adrenal insufficiency. It should also be noted that patients with baseline cortisol ≥18 µg/dL were excluded from this study. A threshold of 18 µg/dL has long been used in clinical practice to exclude adrenal insufficiency. Since improvements in assay methodology are expected to shift this threshold downward, as also suggested by our findings, this exclusion is unlikely to have materially influenced the ROC analysis.

Our study has several limitations. First, it is a single-center, retrospective study with a relatively small sample size. Studies with larger sample sizes, including patients from multiple centers, would help improve the generalizability of the proposed assay-specific cut-off values. Second, we did not simultaneously measure cortisol using both assays for direct comparison. As our study does not directly assess the accuracy between the assays, the results should be interpreted cautiously. In other words, it should be noted that, because the results for the Roche Elecsys Cortisol II group and the Tosoh AIA-CL2400 group were obtained from different populations and during different time periods, the observed differences in cortisol levels cannot be attributed solely to differences in assay methodology. Third, we only assessed the presence or absence of adrenal insufficiency symptoms without detailed categorization. Since symptoms of adrenal insufficiency can vary widely, a more nuanced classification might have been beneficial [3,16-21].

Common symptoms of adrenal insufficiency include non-specific manifestations such as anorexia, nausea, vomiting, abdominal pain, weakness, fatigue, lethargy, fever, confusion, and coma. Fatigue, salt craving, nausea, anorexia, weight loss, and abdominal pain are particularly common in primary adrenal insufficiency, while salt craving and abdominal symptoms are less frequent in secondary adrenal insufficiency. In our study, information about symptoms was extracted from medical records, which limited our ability to classify symptoms in detail. This represents an area for improvement in future studies.

Fourth, data on renin, aldosterone, dehydroepiandrosterone sulfate, and other potentially relevant parameters were not included in our analysis. Some studies suggest that these markers may be useful in distinguishing between primary and secondary adrenal insufficiency [3,22-24]. Similarly, data on sodium levels, eosinophil counts, blood glucose, and hemoglobin were not available for analysis. These parameters should be investigated in future prospective studies.

Fifth, regarding the qualifications of the physicians involved in diagnosing adrenal insufficiency, not all were certified endocrinologists or advanced trainees. However, in such cases, they were required to consult frequently with qualified endocrinologists or supervising physicians. For particularly challenging cases, case conferences were also available. Therefore, we believe that the standard of endocrinological care provided was consistent with Japanese practice norms.

Finally, cases where hydrocortisone replacement was initiated without prior stimulation testing were not included in our study. When multiple symptoms suggestive of adrenal insufficiency are present and baseline cortisol is sufficiently low, stimulation tests are typically not necessary to confirm the diagnosis. For example, if adrenal crisis is suspected, treatment should not be delayed for diagnostic confirmation, and parenteral hydrocortisone therapy should be administered immediately [25]. Therefore, our results should not be interpreted as representing the true prevalence of adrenal insufficiency. Further analysis of the overall prevalence of adrenal insufficiency would be valuable.

Despite these limitations, our study provides rare insights into both the stimulation test results and the diagnosis of adrenal insufficiency in measurement assay by using Roche Elecsys Cortisol II to Tosoh AIA-CL2400 in real clinical practice. Our findings offer meaningful perspectives on the differences in diagnostic cut-offs for adrenal insufficiency between different measurement assays. 

## Conclusions

Our study provides two key messages: First, differences in assay methodology can significantly alter cortisol values and appropriate diagnostic cut-offs, even when transitioning between different monoclonal antibody-based assays. Second, both peak cortisol and the presence of symptoms are crucial for the accurate diagnosis of adrenal insufficiency, with potentially lower cut-off values being appropriate for asymptomatic cases. Although the results suggest that a cut-off value of 17.0 μg/dL is appropriate for the Tosoh AIA-CL2400, the optimal threshold may need to be adjusted depending on the presence or absence of clinical symptoms and/or structural or functional abnormalities of the HPA axis.

Despite its limitations, including the retrospective single-center design, limited sample size, and absence of simultaneous assay comparison, this study offers valuable real-world insights into the impact of assay differences particularly on the diagnosis of patients on the borderline of adrenal insufficiency. We also acknowledge that, in the absence of an independent gold standard assay using a conventional method for this condition, the proposed cut-off values cannot be regarded as absolute diagnostic thresholds; rather, they represent assay-specific, clinically useful reference points to be interpreted alongside clinical judgment. These findings therefore require prospective validation in larger, multi-center cohorts before broader clinical application can be considered.
